# Ileal proteomic changes associated with IL-25-mediated resistance against intestinal trematode infections

**DOI:** 10.1186/s13071-020-04206-y

**Published:** 2020-07-02

**Authors:** María Álvarez-Izquierdo, J. Guillermo Esteban, Carla Muñoz-Antoli, Rafael Toledo

**Affiliations:** Área de Parasitología, Departamento de Farmacia y Tecnología Farmacéutica y Parasitología, Facultad de Farmacia, Universitat de València, Avda. Vicent Andrés Estellés s/n, 46100 Burjassot, Valencia Spain

**Keywords:** Proteomics, Intestine, Interleukin-25, Intestinal helminths, Trematoda, *Echinostoma caproni*

## Abstract

**Background:**

*Echinostoma caproni* (Trematoda: Echinostomatidae) is an intestinal trematode, which has been extensively used to investigate the factors that determine the rejection of intestinal helminths. In this sense, several studies have shown that IL-25 is critical for the development of resistance against *E. caproni* in mice. In fact, treatment of mice with recombinant IL-25 generates resistance against primary *E. caproni* infection. However, the mechanisms by which IL-25 induces resistance remain unknown.

**Methods:**

To study the mechanisms responsible for resistance elicited by IL-25, we analyzed the ileal proteomic changes induced by IL-25 in mice and their potential role in resistance. To this purpose, we compared the protein expression profiles in the ileum of four experimental groups of mice: naïve controls; *E. caproni*-infected mice; rIL-25-treated mice; and rIL-25-treated mice exposed to *E. caproni* metacercariae.

**Results:**

Quantitative comparison by 2D-DIGE showed significant changes in a total of 41 spots. Of these, 40 validated protein spots were identified by mass spectrometry corresponding to 24 proteins.

**Conclusions:**

Our results indicate that resistance to infection is associated with the maintenance of the intestinal epithelial homeostasis and the regulation of proliferation and cell death. These results provide new insights into the proteins involved in the regulation of tissue homeostasis after intestinal infection and its transcendence in resistance.
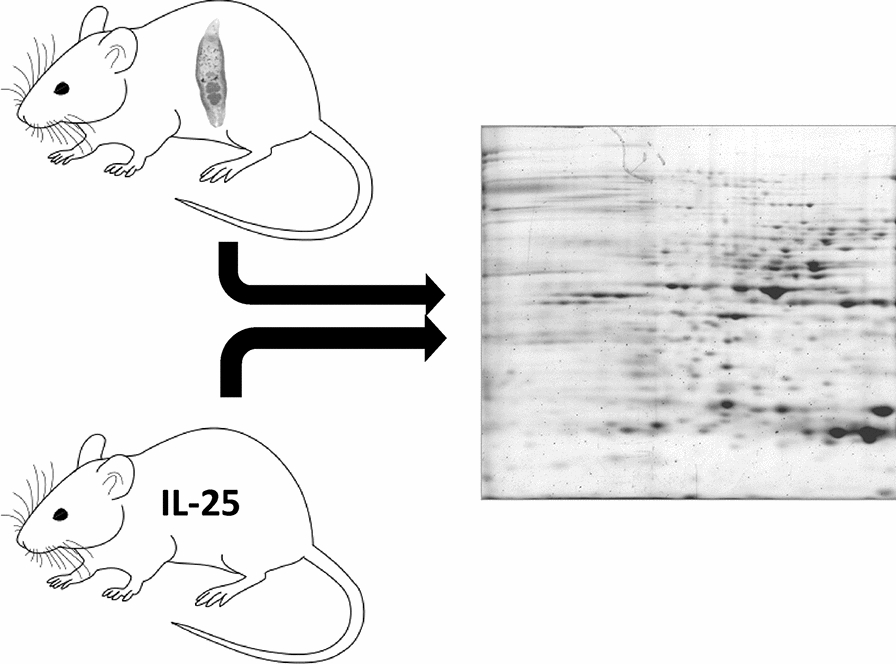

## Background

Intestinal helminth infections affect more than one billion people worldwide, mainly in developing regions of Asia, Africa and Latin America [1]. These infections cause high morbidity, with most common symptoms related to effects on nutrition inducing malabsorption syndrome, vitamin deficiencies, growth retardation or impaired cognitive function among other disorders. Moreover, other complications such as intestinal obstruction, chronic dysentery, rectal prolapse, anemia or debilitating disease may appear [2, 3]. Apart from their importance for human health, helminth infections are a relevant cause of economic losses in livestock, both by decreased productivity, an also in relation to the indirect costs of anthelmintic treatments [4]. Intestinal helminth infections caused by trematodes constitute a major group affecting both humans and animals [5]. Intestinal trematodes are a large group of parasites and about seven million people are infected worldwide [6]. About 76 species belonging to the family Echinostomatidae have been reported to infect humans [6]. Human infection occurs as a consequence of eating raw or undercooked foods containing infective metacercariae. High incidence of intestinal trematodiasis is strongly associated with populations living near freshwater bodies and the practice of eating raw or undercooked aquatic products [5]. One of the most relevant group of trematodes causing human infections, mainly in East and Southeast Asia, are the members of the family Echinostomatidae. Echinostomes are cosmopolitan parasites that infect a large number of different warm-blooded hosts. More than 20 species of *Echinostoma* are known to cause human infections worldwide [5]. Moreover, members of the genus *Echinostoma*, and particularly *Echinostoma caproni*, have been widely used as experimental models to study helminth-vertebrate host relationships, especially in relation to the factors that determine the resistance to intestinal helminth infections. *Echinostoma caproni* is an intestinal trematode without tissue migration within the definitive host. The metacercariae excyst in the duodenum and the excysted worms migrate to the ileum and attach to the mucosa [7]. *Echinostoma caproni* has a wide range of definitive hosts, although its compatibility differs considerably between rodent species due to different worm survival and development in each host species [7]. In mice, the infection becomes chronic, while in hosts of low compatibility, such as rats, the worms are rapidly rejected in a few weeks post-infection (wpi) [8, 9].

In recent years, IL-25 is considered a crucial cytokine in resistance to intestinal helminths. IL-25 induces Th2 immunity and facilitates anti-inflammatory functions *via* the downregulation of Th17 and Th1 responses [10–12]. Expression of IL-25 induces resistance to gastrointestinal helminth infections due to the activation of Th2 responses that mediate effector mechanisms (including mast cell hyperplasia, smooth muscle hypercontractility, expression of RELM-β, and intestinal mastocytosis, among others) for parasite expulsion [13]. Intestinal tuft cells are the main source of IL-25 and release IL-25 upon helminth establishment. Subsequently, group 2 of innate lymphoid cells (ILC2) produce large amounts of IL-13 activating dendritic cells in the lamina propria and enhancing their migration to mesenteric lymph nodes to polarize naïve CD4+ T cells into Th2. ILC2 and basophils can also perform antigen presentation to CD4+ T cells and induce Th2 polarization. Th2-polarized cells release an array of cytokines and expand themselves through positive feedback loops, amplifying the response and enhancing resistance to infection [13].

Previous studies of our group showed that IL-25 is crucial for resistance to *E. caproni* and the susceptibility of mice relies on the inability of this host species to produce IL-25 in response to infection [14, 15]. Susceptibility of mice to primary *E. caproni* infection was associated with low expression of intestinal IL-25, whilst deworming *via* administration of praziquantel (pzq) was accompanied by an increase in IL-25 production and, subsequently, the development of a Th2-type phenotype preventing the establishment of secondary infections [14, 15]. However, little is known about the mechanism by which IL-25 generates resistance against intestinal helminths. In the present study, we analyzed the changes in the production of proteins induced by IL-25 in the ileum of mice that may be implicated in the generation of resistance against intestinal helminths.

## Methods

### Animal and infection procedures

The present study was achieved using a total of 15 male ICR (CD1) mice weighing 30–35 g. The *E. caproni* strain and the infection procedures carried out have been previously described [9, 16]. Briefly, encysted metacercariae were removed from kidneys and pericardium of experimentally infected *Biomphalaria glabrata* snails and used for infection. Mice were randomly allocated into 4 groups (3 mice in each group). Animals belonging to one of the groups were infected by gastric gavage with 50 metacercariae of *E. caproni*. Mice of the second group were treated with rIL-25 (R&D Systems^®^, Minneapolis, USA) (concentration: 0.2 µg/µl each) in 150 µl of PBS during each of the 4 days prior to infection with 50 metacercariae of *E. caproni* as described above. Mice of the third group were simply treated with penicillin under the conditions described above. This group was not exposed to metacercariae of *E. caproni*. Finally, the remaining 5 mice were used as a control and were not exposed to rIL-25 or *E. caproni* metacercariae. All mice were necropsied one week after the experimental infection of the animals belonging to the first and second groups of mice. The animals were maintained under standard conditions with food and water *ad libitum*.

### Intestinal epithelial cell isolation and protein extraction

Ileal sections from mice in each group were removed at necropsy and intestinal epithelial cells (IECs) were isolated. Briefly, the intestinal sections were opened longitudinally and rinsed by shaking in washing buffer: ice-cold Hank’s balanced salt solution (HBSS) containing 2% heat-inactivated fetal calf serum (FCS). Supernatant was then removed, and fresh washing buffer was added to the ileal sections. This procedure was repeated 4 times, until the supernatant was clear. The tissue was cut into small, 1 cm long, segments and incubated for 20 min at 37 °C in HBSS containing 10% FCS, 1 nM EDTA, 1 mM DTT, 100 U/ml penicillin and 100 µg/ml streptomycin (dissociation buffer). The supernatant was collected and maintained on ice and the incubation was repeated a second time with fresh dissociation buffer. Supernatants were combined and filtered through a 100 nm cell strainer before IECs were pelleted by centrifuging at 200× *g* for 10 min at 4 °C and washed three times in PBS under the same centrifuge conditions to remove any residual medium.

Protein extraction was performed using M-PER Mammalian Protein Extraction Reagent (Thermo Fisher Scientific, Waltham, USA) according to the manufacturer’s instructions. Extraction reagent was added to the IECs pellet (20:1, v/v), vortexed to mix and incubated at room temperature (RT) for 20 min under continuous gentle agitation. The lysate was then clarified by centrifugation at 18,000×* g* for 15 min at 4 °C, transferred into a new tube, and stored at − 80 °C until use.

### Preparation of biological replicates and protein labeling

To increase the biological significance avoiding erroneous conclusions related to individual variations, 4 biological replicates were prepared for each experimental group: infected with *E. caproni*; rIL-25-treated mice exposed to *E. caproni* metacercariae; rIL-25-treated mice; and naïve animals.

Three of these replicates were obtained from different animals and the fourth was obtained by mixing the previous three by applying the same amount of protein from each sample (20 μg/sample) and included to increase the power of the analysis [17]. Then, 200 μg of protein from each biological replicate was cleaned and precipitated using 2-D Clean-Up kit (GE Healthcare, Chicago, USA) to remove salts and other substances that interfere with labeling and electrophoresis. The samples were resuspended in DIGE tagged buffer (7 M urea, 2 M tiourea, 4 % CHAPS and 20 mM Tris). The protein concentration after precipitation was determined using the RC DC (Bio-Rad Protein Assay; Bio-Rad, Hercules, USA) method, using BSA as standard protein. The concentrations for labeling with fluorochromes should be between 1–20 μg/μl, with optimum concentration for labeling according to the manufacturer’s instructions being between 5–10 μg/μl. With the precipitated samples, 100 μg pools needed for the experiment were made for each group, with equimolar amounts of each sample in each group and quantified again. The DIGE experiment was designed to perform 8 gels containing the samples of the four groups to be compared. After checking that the pH of all samples was between 8–8.5, CyDye DIGE Fluor (GE Healthcare^®^) fluorochromes were labeled according to the protocol recommended by the manufacturer. One microliter of dye (400 pmol) was added to each sample and maintained on ice for 30 min in the dark. The reaction was stopped by adding 1 µl of 10 mM lysine. To minimize any dye-specific labeling artefacts, 2 biological replicates of each experimental group were labeled with Cy3 and the other 2 were labeled with Cy5. The internal standard, prepared by mixing the same amount of protein of each sample included in the experiment, was always labeled with Cy2.

### 2D differential in gel electrophoresis (2D-DIGE)

To analyze the effect of IL-25 in the course of *E. caproni* infection, ileal protein extracts from naïve, infected, rIL-25-treated and rIL-25-treated and exposed to metacercariae mice were compared over 8 2D-DIGE to analyse changes in the intestinal production of proteins. The 8 pairs of Cy3- and Cy5-labeled biological replicates (50 µg of protein each) were combined with a 50 µg aliquot of the Cy2-labeled internal standard. The mixtures containing 150 µg of protein were then separated in the first dimension, i.e. isoelectric focusing, and the second dimension, i.e. molecular weight. The IPG strips (24 cm, non-linear pH 3–11) were rehydrated overnight with rehydration buffer (8 M urea, 4% CHAPS, 1% ampholytes and 12 µl/ml of DeStreak™ (Merck, St. Louis, USA), and the labeled samples were then applied to the strips by anodic cup loading, after the addition of DTT and ampholytes up to a final concentration of 65 mM and 1%, respectively. Isoelectric focusing was carried out at 20 °C in the Ettan IPGphor 3 System (GE Healthcare) as follows: (i) 300 V for 4 h; (ii) gradient to 1000 V for 6 h; (iii) gradient to 8000 V for 3 h; and (iv) 8000 V up to 32,000 Vh. Prior to the second dimension the strips were equilibrated in two steps, 15 min each, in equilibration buffer (50 mM Tris, 6 M urea, 30% glycerol and 2% SDS) containing either 2% DTT or 2.5% iodoacetamide, respectively. The separation of proteins in the second dimension was performed on an Ettan DALTsix system (GE Healthcare) using 12.5% polyacrylamide gels. Electrophoresis was run at 1 W/gel for 1 h followed by 5 h, approximately, at 15 W/gel.

### Imaging and 2D-DIGE analysis

Gels were scanned in a Typhoon™ 9400 Variable Mode Imager (GE Healthcare) at appropriate wavelengths for each fluorophore at 50 µm resolution: Cy2 (488/520 nm); Cy3 (532/580 nm); and Cy5 (633/670 nm). The irrelevant information was removed using ImageQuant Tools software and DeCyder v7.0 software (Applied Biomics Inc., Hayward, USA) was used for image analysis. The differential in gel analysis module was employed for automatic spot detection and abundance measurements in each individual gel, comparing the normalized volume ratio of each spot from a Cy3- or Cy5-labeled sample to the corresponding Cy2 signal from the internal standard. Datasets were collectively analyzed by means of the biological variation analysis module of the same software, allowing inter-gel matching and calculation of standardized average volume ratios (AVRs) for each protein spot over all the gels that comprised the study. Statistical analysis was carried out for each alteration in AVR using one-away ANOVA, together with the corresponding *post-hoc* analysis (Bonferroniʼs *post-hoc* t-test), and the false discovery rate (FDR) test, which avoids the introduction of false positives when performing multiple comparisons. FDR test determines adjusted *P*-values for each test and controls the number of false discoveries in those tests that result in a discovery. Statistical significance was considered when *P* < 0.01 and *q* < 0.05 in the ANOVA and FDR analyses, respectively. Moreover, inter-gel matching of statistically different spots was manually confirmed.

Unsupervised principal components analyses (PCA) and hierarchical cluster analyses (HCA) (based on Euclidean distance) were performed using the DeCyder extended data analysis module, both on all protein spots present at least in 7 of the 8 gels of the experiment (85% presence) and the set of spots that were found to be significantly differentially expressed among the groups compared. These multivariate analyses clustered the individual biological replicates based on a collective comparison of expression patterns from the set of proteins chosen, without any *a priori* knowledge of the biological reasons for clustering [17–20].

### LC-MS/MS and protein identification

Spots showing significant changes in protein abundance among groups were manually excised from the gel and washed twice with double-distilled water. Thereafter, proteins were reduced in 100 mM ammonium bicarbonate containing 10 mM DTT for 30 min at 56 °C, alkylated with iodoacetamide 55 mM in 100 mM ammonium bicarbonate for 20 min at RT in the dark and, finally, digested in-gel with an excess of sequencing grade trypsin (Promega, Madison, USA) overnight at 37 °C, as described before [21]. Protein digestion was stopped with 1% trifluoroacetic acid (TFA) and peptides were dried in a vacuum centrifuge and resuspended in 7 μl of 0.1% TFA, pH 2. Following, liquid chromatography and tandem mass spectrometry (LC-MS/MS) was performed for protein identification. Five microliters of each sample was loaded onto a trap column: NanoLC Column, 3 μ C18-CL, 350 μm × 0.5 mm (Eksigent, AB Sciex, Old Connecticut Path, USA) and desalted with 0.1% TFA at 3 μl/min for 5 min. The peptides were then loaded onto an analytical column: LC Column, 3 μ C18-CL, 75 μm × 12 cm (Nikkyo Technos, Tokyo, Japan), equilibrated with 5% acetonitrile (ACN) and 0.1% formic acid (FA). Elution was carried out with a gradient of 5–40% B in A for 15 min (A: 0.1% FA; B: ACN, 0.1% FA) at a constant flow rate of 300 nl/min. Peptides were analyzed in a mass spectrometer nanoESI qQTOF (5600 TripleTOF; AB Sciex, Old Connecticut Path, USA).

The sample was ionized applying 2.8 kV to the spray emitter. Analysis was carried out in a data-dependent mode. Survey MS1 scans were acquired from 350–1250 m/z for 250 ms. The quadrupole resolution was set to ‘UNIT’ for MS2 experiments, which were acquired 100–1500 m/z for 50 ms in the ‘high sensitivity’ mode. The following switch criteria were used: charge, 2+ to 5+; minimum intensity; 70 counts per second (cps). Up to 50 ions were selected for fragmentation after each survey scan. Dynamic exclusion was set to 15 s. The system sensitivity was controlled with 2 fmol of 6 proteins (LC Packings, Thermo Fisher Scientific, San Diego, USA).

### Database search

Database search was carried out using the ProteinPilot v5.0. search engine (AB Sciex). ProteinPilot default parameters were employed to generate a peak list directly from 5600 TripleTof wiff files and the Paragon algorithm of ProteinPilot v5.0 was used to search in Uniprot database (version 01-2017; https://www.uniprot.org) with the following parameters: trypsin specificity; cys-alkylation; taxonomy restricted to mice; and the search effort set to through. Identifications were considered positive when there were at least two different matching peptides (≥ 95% confidence) and the ProteinPilot unused score was > 1.3, which means that proteins are identified with confidence ≥ 95%. Functional annotation was performed using the Uniprot database.

## Results

### Experimental infection with metacercariae of *E. caproni* and worm recovery

All the rIL-25-treated mice exposed to metacercariae were negative to infection at necropsy. In contrast, all the non-treated mice exposed to metacercariae became positive to infection and the percentage of worms recovered ranged between 40–100% (mean 69.36 ± 16.29%).

### Analysis of protein production profiles by 2D-DIGE

The 2D-DIGE proteomic analysis was implemented on whole ileal cell extracts in a total of 12 replicates, corresponding to 4 experimental groups (3 replicates each) referred as: control, rIL-25-treated mice; rIL-25-treated mice exposed to metacercariae; and infected mice. 2D-images were analyzed using the DeCyder software and both multivariate and univariate analysis were applied to identify: (i) the similarity in intestinal protein production profiles among experimental groups; and (ii) particular differences in protein abundance between each group with respect to the others (Additional file 1: Figure S1, Additional file 2: Figure S2, Additional file 3: Figure S3).

The inter-gel spot matching revealed 172 well defined spots with 85% of presence, found in at least 7 of the 8 gels that covered the experiment. The average abundance of each spot among the 24 images of our study was calculated, and significant differences were considered when *P* < 0.01, both in one-way ANOVA and in the *post-hoc* analyses.

### Multivariate statistics: principal component and cluster analysis

**Fig. 1:**
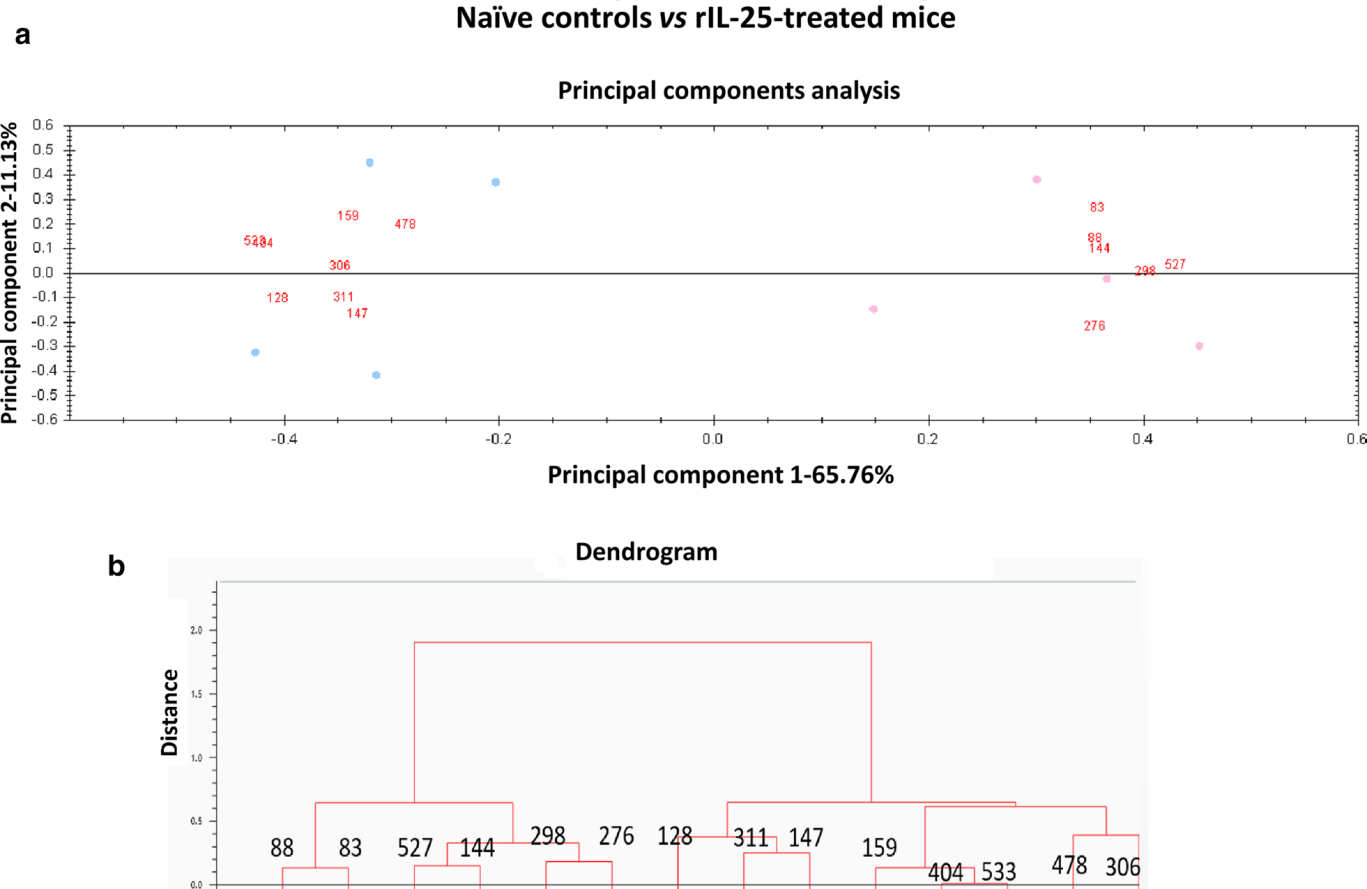
Multivariate statistical analysis applied to the set of 41 manually validated differential spots (85% of presence; *P* < 0.01; *q* < 0.05) in the 2D-DIGE experiment comparing naïve controls and rIL-25-treated mice. **a** Plot from the principal components analysis between compared groups separated in two areas according to their overexpression in one group in relation to the other. **b** Dendrogram from the hierarchical cluster analysis based on Euclidean distance

**Fig. 2:**
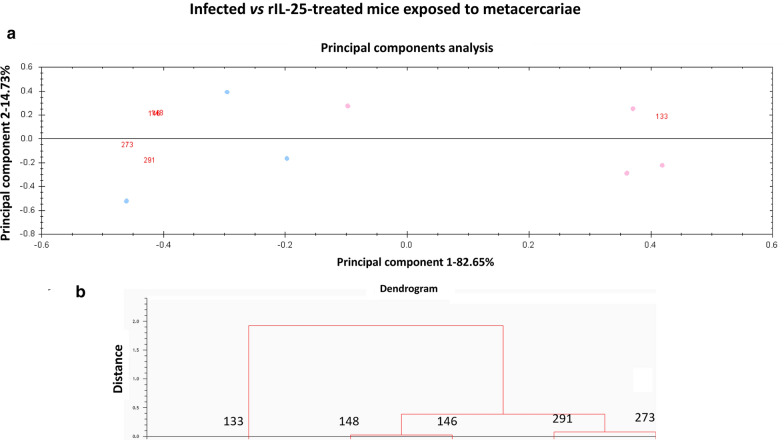
Multivariate statistical analysis applied to the set of 41 manually validated differential spots (85% of presence; *P* < 0.01; *q* < 0.05) in the 2D-DIGE experiment comparing infected *vs* rIL-25-treated mice exposed to *Echinostoma caproni* metacercariae. **a** Plot from the principal components analysis between compared groups separated in two areas according to their overexpression in one group in relation to the other. **b** Dendrogram from the hierarchical cluster analysis based on Euclidean distance)

**Fig. 3:**
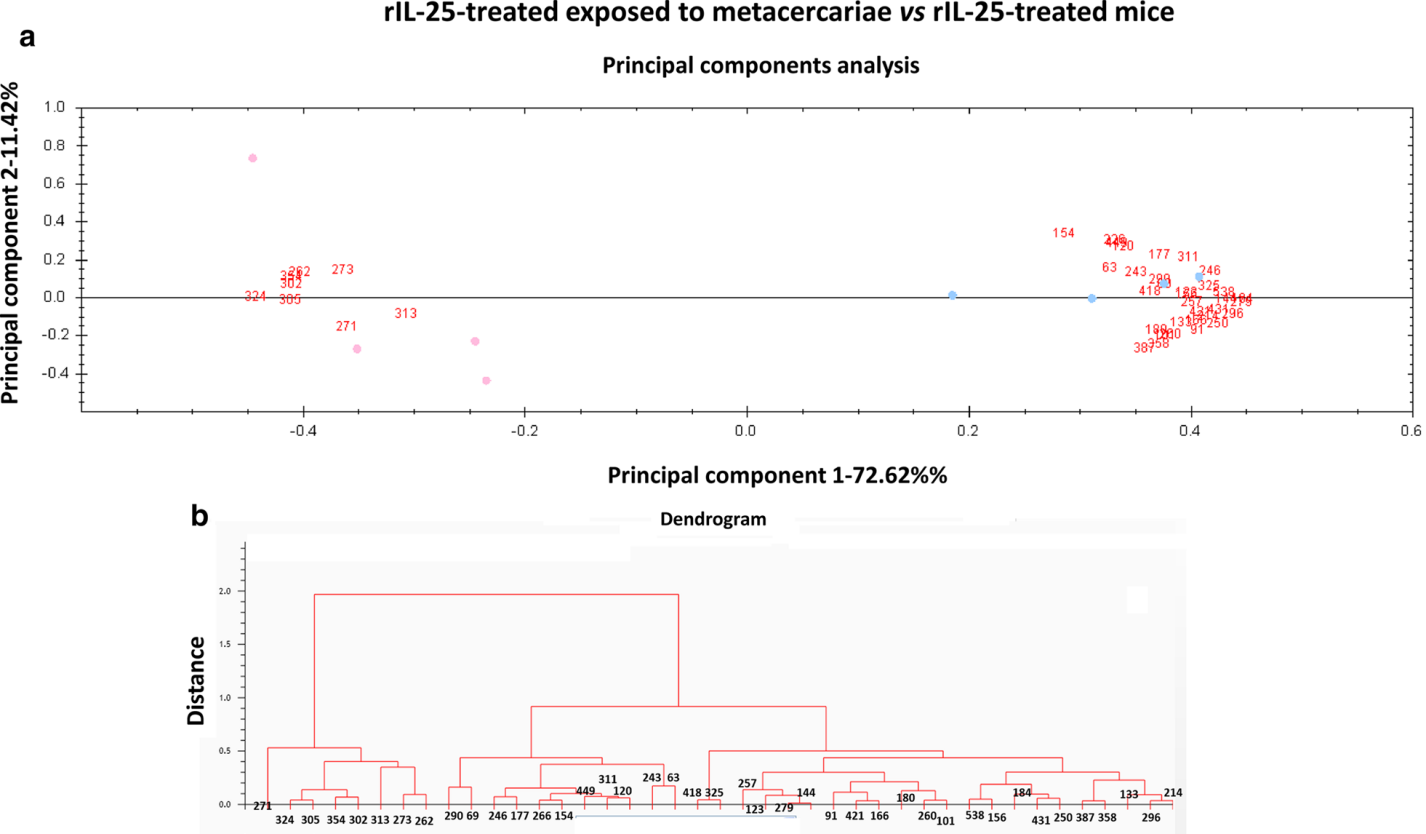
Multivariate statistical analysis applied to the set of 41 manually validated differential spots (85% of presence; *P* < 0.01; *q* < 0.05) in the 2D-DIGE experiment comparing rIL-25-treated exposed to *Echinostoma caproni* metacercariae *vs* rIL-25-treated mice. **a** Plot from the principal components analysis between compared groups separated in two areas according to their overexpression in one group in relation to the other. **b** Dendrogram from the hierarchical cluster analysis based on Euclidean distance

PCA and CAs between groups were performed on the 172 spots with 85% of presence in the experiment and the 41 validated spots displaying significant differences among groups, with *P* < 0.01 in one-way ANOVA. In the three cases, both PCA and CAs were compared two by two: (i) including biological replicates from control and rIL25-treated mice; (ii) including biological replicates from rIL25-treated mice and rIL25-treated mice exposed to metacercariae; and (iii) including biological replicates from infected and rIL25-treated mice exposed to metacercariae. In the PCA, data clustered according the group mice, spots with greater presence in each group of mice with respect to the other appeared highlighted. Likewise, CAs grouped the spots according to how similar their expression profile was between compared experimental groups. Hence, following the results of the multivariate statistical analyses, the 4 experimental groups were used as 3 comparative pairs due to their importance to analyze the role of IL-25: (i) infected animals *vs* rIL-25-treated animals; (ii) rIL-25-treated mice exposed to metacercariae *vs* rIL-25-treated animals; and (iii) naïve controls *vs* rIL-25-treated mice (Figs. 1, 2, 3).

### One-way ANOVA and *post-hoc* analysis

**Fig. 4:**
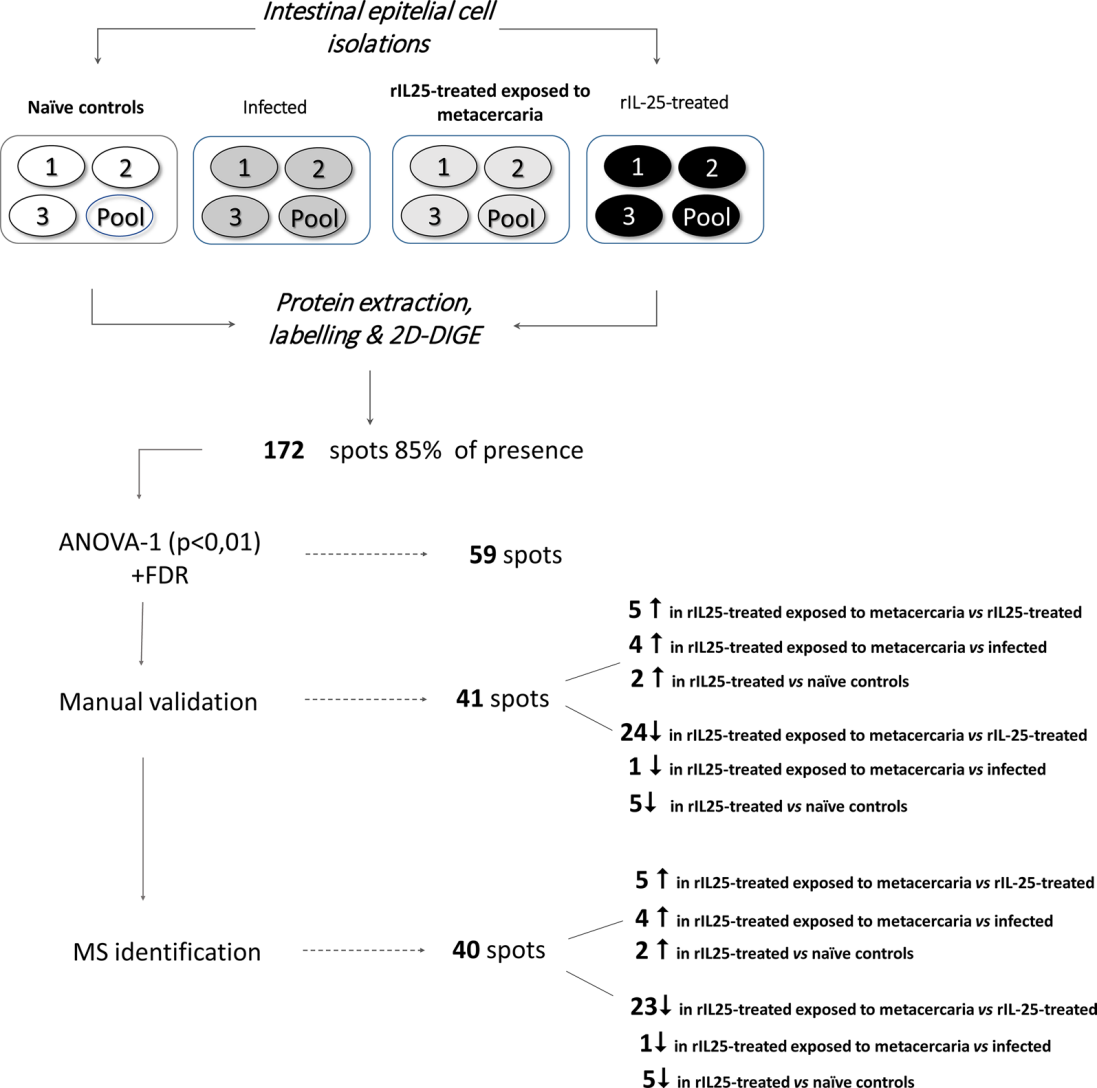
Schematic overview of the results obtained by 2D-DIGE in the comparison of protein production profiles of intestinal epithelial cells isolated from naïve controls, rIL25-treated mice, infected animals and rIL25-treated mice exposed to metacercariae of *Echinostoma caproni*

A total of 59 differentially expressed spots (34.3%) were found displaying *q* < 0.05 in the FDR test. To guarantee the accurate comparison of spots among gels, the correspondence of these 59 spots were manually validated through all the gels, and 41 were unequivocally confirmed (Fig. 4, Additional file 1: Figure S1, Additional file 2: Figure S2, Additional file 3: Figure S3).

Differentially identified spots were up- or downregulated (5 and 24, respectively) in the ileum of rIL-25-treated mice with respect rIL-25-treated animals and infected mice (4 and 1, respectively). Moreover, we found 7 spots differentially identified between naïve controls and rIL-25-treated mice from which 2 of them were upregulated and the remainder 5 spots were downregulated. Further details of the computational comparison of differential spots are shown in Additional file 4: Table S1 for non-similar groups (i.e. naïve controls *vs* rIL-25-treated animals exposed to metacercariae and rIL-25-treated mice *vs* infected animals).

### Identification of differentially produced proteins

We accurately identified by MS and database search a total of 40 validated spots (5 upregulated and 24 downregulated in rIL-25-treated animals exposed to metacercariae *vs* rIL-25-treated mice; 4 upregulated and 1 downregulated in rIL-25-treated animals exposed to metacercariae *vs* infected mice; 2 upregulated and 5 downregulated in rIL-25-treated mice *vs* naïve animals) (Fig. 4). They correspond to a total of 24 different proteins, since 6 of these proteins were identified in more than 1 protein spot. These redundancies appear to be related to different post-translational modifications, different isoforms (differentiated on the basis of molecular weight or isoelectric point) or to protein modifications during the preparation of the samples. Identified proteins are classified in Tables 1, 2, 3 according to their function, indicating for each spot the up- or downregulation in relation to inoculation of rIL25 in presence and/or absence of *E. caproni* infection in mice. Differentially expressed proteins were classified in: metabolic enzymes; structural proteins; antioxidant and detoxifying enzymes; calcium-binding proteins; and cell regulation proteins.

**Table 1:** Proteins identified by 2D-DIGE/mass spectrometry as differentially expressed between intestinal epithelial cells of infected mice *versus* rIL-25-treated animals and exposed to metacerariae of *Echinostoma caproni*

Spot	Protein	Species	Expression	MW (kDa) Expected/Observed	Isoelectric point Expected/Observed	Cellular role	Location	Score	Coverage (%)	Peptides
133	Enolase 1B	*Mus musculus*	− 1.4	47/116	6.37/6.37	Glycolysis	Cyt	45.44	66.59	8
						Plasminogen activation	PM			
						Ornithine metabolism	Mit			
146	Enolase 1B	*Mus musculus*	+ 1.9	47/112	6.37/7.17	Glycolyis	Cyt	38.54	45.16	112
						Plasminogen activation	PM			
						Ornithine metabolism	Mit			
148	Enolase 1B	*Mus musculus*	+ 2.0	47/111	6.37/6.67	Glicolisis	Cyt	108.86	87.33	176
						Plasminogen activation	PM			
						Ornithine metabolism	Mit			
273	Glyceraldehyde-3-phosphate dehydrogenase	*Mus musculus*	+ 2.4	35/93	8.44/8.44	Glycolysis	Cyt	32.38	62.76	29
291	Lactate dehydrogenase	*Mus musculus*	+ 1.5	36/88.8	7.61/9.26	Pyruvate fermentation to lactate	Cyt	16.31	45.48	29

*Abbreviations*: MW, molecular weight; Cyt, cytoplasmatic; PM, plasma membrane

**Table 2:** Proteins identified by 2D-DIGE/mass spectrometry as differentially expressed between intestinal epithelial cells of rIL-25-treated mice *versus* rIL-25-treated animals and exposed to metacerariae of *Echinostoma caproni*

Spot	Protein	Species	Expression	MW (kDa) Expected/Observed	Isoelectric point Expected/Observed	Cellular role	Location	Score	Coverage (%)	Peptides
Metabolic enzymes										
63	Pyruvate kinase PKM	*Mus musculus*	− 1.9	58/132	7.17/8.20	Glycolysis	Cyt; Nuc	51.34	52.54	30
69	Pyruvate kinase PKM	*Mus musculus*	− 1.7	58/132	7.17/8.48	Glycolysis	Cyt; Nuc	46.03	42.94	26
101	Glutamate dehydrogenase 1	*Mus musculus*	− 1.6	61/126	8.05/8.05	Glutamine anaplerosis	Mit	70.19	62.54	26
123	6-Phodphogluconate dehydrogenase	*Mus musculus*	− 1.7	53/122	6.81/7.62	Pentose phosphate pathway	Cyt	14	18.84	65
144	Enolase 1B	*Mus musculus*	− 1.8	47/118	6.37/8.46	Glycolysis	Cyt; PM; Mit	89.91	79.95	30
154	Fumarate hydratase	*Mus musculus*	− 1.6	54/118	9.12/9.12	Krebs cycle	Mit	39.41	35.5	25
156	Enolase 1B	*Mus musculus*	− 1.8	47/118	6.37/6.37	Glycolysis	Cyt; PM; Mit	46.26	51.15	35
166	Creatine kinase B-type	*Mus musculus*	− 2.0	43/116	5.34/5.34	Cretine kinase activity	Cyt	12.4	29.92	7
184	Phosphoglycerate kinase 1	*Mus musculus*	− 2.3	45/114	8.02/9.5	Glycolysis	Cyt	46.01	63.55	39
214	Glyceraldehyde-3-phosphate dehydrogenase	*Mus musculus*	− 1.9	36/108	8.44/9.43	Glycolysis	Cyt	15.03	42.94	8
226	Aspartate aminotransferase	*Mus musculus*	− 2.2	47/108	9.13/9.92	Aminoacid metabolism	Mit	48.42	50	39
250	Transaldolase	*Mus musculus*	− 1.8	42/102	6.57/7.04	Pentose phosphate pathway	Cyt	20.35	35.34	16
257	Malate dehydrogenase	*Mus musculus*	− 1.8	37/96	6.16/6.16	Krebs cycle	Cyt	28.05	41.62	19
262	Glyceraldehyde-3-phosphate dehydrogenase	*Mus musculus*	+ 1.8	36/100	8.44/8.44	Glycolysis	Cyt	13.22	39.64	11
279	Ornithine carbamoyltransferase	*Mus musculus*	− 2.0	39/100	8.81/8.30	Ornithine metabolism	Mit	35.79	45.87	55
290	Palmitoyl-protein thioesterase	*Mus musculus*	− 2.6	35/94	8.26/8.85	Palmitoyl metabolism	Cyt	10,04	21.5	5
296	Malate dehydrogenase	*Mus musculus*	− 1.8	37/94	6.16/6.28	Krebs cycle; malate shuttle	Cyt	61.69	74.55	70
302	Malate dehydrogenase	*Mus musculus*	+ 2.2	37/90	6.16/5.58	Krebs cycle; malate shuttle	Cyt	17.01	36.53	10
305	Malate dehydrogenase	*Mus musculus*	+ 1.7	37/90	6.16/5.98	Krebs cycle; malate shuttle	Cyt	42.04	53.59	45
387	Triosephosphate isomerase	*Mus musculus*	− 1.8	32/60	5.56/8.66	Glycolysis; gluconeogenesis	Cyt	3.9	16.03	3
Structural proteins										
243	Junction plakoglobin	*Mus musculus*	− 2.3	82/104	5.75/6.43	Cell adhesión	Cyt	39.85	46.98	20
260	Junction plakoglobin	*Mus musculus*	− 1.5	82/102	5.75/8.18	Cell adhesion	Cyt	29.38	29.4	18
325	Junction plakoglobin	*Mus musculus*	− 1.6	82/86	5.75/6.34	Cell adhesion	Cyt	12.5	17.85	8
Antioxidant-detoxifying enzymes										
358	Dihydropteridine reductase	*Mus musculus*	− 2.5	22/74	7.67/8.40	Oxireductase activity	Mit	11.8	31.92	8
418	Glutathione S-transferase P 1	*Mus musculus*	− 1.6	24/60	7.69/8.33	Glutathione conjugation and detoxification	Cyt; Mit; Nuc	18.06	57.14	35
421	Glutathione S-transferase P 1	*Mus musculus*	− 1.7	24/59	7.69/9.21	Glutathione conjugation and detoxification	Cyt; Mit; Nuc	31.38	78.1	75
431	Peroxiredoxin-1	*Mus musculus*	− 2.0	22/57	8.26/8.26	Redox regulation	Cyt	10.18	33.67	7
Calcium-binding proteins										
271	Annexin A2	*Mus musculus*	+ 1.6	39/101	7.55/8.84	Membrane transport; fibrin homeostasis	PM	39.75	71.98	18
324	Annexin A4	*Mus musculus*	+ 2.5	36/86	5.43/5.02	Membrane transport	PM ext	23.72	41.69	15

*Abbreviations*: MW, molecular weight; Cyt, cytoplasmatic; PM, plasma membrane; Mit, mitochondrion; Nuc, nucleus

**Table 3:** Proteins identified by 2D-DIGE/mass spectrometry as differentially expressed between intestinal epithelial cells of naïve control mice *versus* rIL-25-treated animals

Spot	Protein	Species	Expression	MW (kDa) Expected/Observed	Isoelectric point Expected/Observed	Cellular role	Location	Score	Coverage (%)	Peptides
Metabolic enzymes										
144	Enolase 1B	*Mus musculus*	+ 1.2	47/110	6.37/6.37	Glycolysis	Cyt	89.91	75.81	112
						Plasminogen activation	PM			
						Ornithine metabolism	Mit			
147	Enolase 1B	*Mus musculus*	− 1.5	47/110	6.37/6.75	Glycolysis	Cyt	37.45	48.62	21
						Plasminogen activation	PM			
						Ornithine metabolism	Mit			
533	Triosephosphate isomerase	*Mus musculus*	− 1.9	32/60	5.56/6.28	Glycolysis	Cyt	25	45.48	16
						Gluconeogenesis				
Structural proteins										
527	Junction plakoglobin	*Mus musculus*	+ 1.7	82/27	5.75/8.70	Cell adhesion	Cyt	3.33	3.44	4
404	Peroxiredosin-4	*Mus musculus*	− 1.9	31/60	6.67/5.90	Redox regulation	Cyt	10.61	42.34	8
Cell regulation proteins										
159	Proliferation-associated 2G4	*Mus musculus*	− 1.8	50/108	6.41/6.83	Apoptotic process	Cyt	29.09	41.62	16
306	Receptor of activated protein C kinase 1	*Mus musculus*	− 1.8	35/83	7.6	Apoptotic process	PM	13.62	27.76	7
						Biological rhythms	Nuc			
						Translation regulation				

*Abbreviations*: MW, molecular weight; Cyt, cytoplasmatic; PM, plasma membrane; Mit, mitochondrion; Nuc, nucleus

## Discussion

Recent studies of our group have shown that IL-25 is crucial in resistance against *E. caproni* secondary infections. Susceptibility to primary infections was associated with low levels of intestinal IL-25 expression, whilst deworming by treatment with praziquantel induced a sudden increase in IL-25 expression preventing the establishment of secondary infections [14, 15]. However, the role of IL-25 in resistance to infection is not well defined. Herein, we analyze the proteomic changes induced by IL-25 that may contribute to resistance to infection.

Resistance to *E. caproni* infection has been associated with the preservation of the intestinal homeostasis despite the possible damage induced by the parasite. In resistant hosts, *E. caproni* infection elicits a rapid renewal of the intestinal epithelium to maintain the intestinal homeostasis, impairing the proper worm establishment. In contrast, in susceptible hosts, such as mice, the establishment of chronic infections is related to the disruption of the intestinal homeostasis causing tissue hyperplasia [17, 19, 20]. Although mice are susceptible hosts, treatment with rIL-25 prior to infection induces complete resistance to the infection [15]. Our results support that IL-25 may contribute to resistance by the enhancement of intestinal homeostasis *via* activation of the canonical wingless-related integrator site (Wnt)/β-catenin signaling pathway. Treatment of naïve mice with rIL-25 only elicited changes in the production of a total of five proteins, including the structural protein junction plakoglobin or β-catenin. This protein is a member of the catenin family, paralog of β-catenin, and is a component of desmosomes. It is involved in the mechanisms of cell adhesion and is essential to maintain and regulate intestinal epithelial homeostasis [22–24]. Plakoglobin participates in the canonical pathway of Wnt/β-catenin. Elevated levels of plakoglin promote the stabilization and nuclear localization of β-catenin enhancing the activation of Wnt/β-catenin signaling, and activation of this pathway is essential for the maintenance of intestinal homeostasis [25, 26]. Wnt signaling activation is dependent on the nuclear translocation of β-catenin. The intracellular accumulation of non-phosphorylated β-catenin induces its translocation to the nucleus and the consequent activation of the T-cell factor/lymphocyte enhancer factor transcription factor families to regulate gene transcription [27]. Plakoglobin participates in the canonical pathway of Wnt/β-catenin signaling since this protein inhibits the glycogen synthase kinase (GSK3β)-mediated nuclear localization of β-catenin. GSK-3β is a relevant member since it regulates the Wnt/β-catenin target gene expression by controlling the level of cytoplasmic β-catenin and its nuclear traslocation [28]. Elevated levels of plakoglin facilitate the stabilization and nuclear localization of β-catenin [25] and may enhance intestinal homeostasis despite the damage caused by the infection. Oudhoff et al. [29] reported that Wnt/β-catenin signaling is an important component of resistance to the intestinal nematode *Trichuris muris* in mice. These authors showed that Wnt expression programs are induced upon infection with *T. muris* eggs and wild type mice were able to expel the infection. In contrast, mice deficient in SETD7 (a member of the suppressor of variegation 3-9-Enhancer of zeste-Trithorax domain-containing family of lysine methyltransferases) were not able to reject the infection. SETD7 controls IEC turn over by the modulation of the developmental signaling pathway Wnt/β-catenin. Lack of SETD7 resulted in downregulation of Wnt/β-catenin and susceptibility to infection [29]. The fact that exposure of rIL-25-treated mice to *E. caproni* metacercariae induced a significant downregulation of three isoforms of plakoglobin with respect to rIL-25-treated mice supports that plakoglobin plays a major role in *E. caproni* infections, and its potential role in resistance to infection.

Strikingly, two other proteins involved in cell differentiation and tissue homeostasis also became altered by the treatment with rIL-25. Proliferation-associated 2G4 (PA2G4) and a receptor of activated protein C kinase 1 (RACK1) were found to be downregulated in rIL-25-treated mice with respect to naïve mice. PA2G4, also known as EBP1, is an RNA-binding protein implicated in growth regulation. This protein participates in pre-ribosomal ribonucleoprotein complexes and is involved in ribosome assembly and the regulation of intermediate and late steps of rRNA processing. EBP1 interacts with the cytoplasmic domain of the ErbB3 receptor contributing to the transduction of growth regulatory signals. This protein also acts as a transcriptional corepressor of androgen receptor-regulated genes and other cell cycle regulatory genes *via* its interactions with histone deacetylases. Furthermore, EBP1 is involved in growth inhibition [30, 31]. The EBP1-binding in promoters regulated by E2F can result in an improved ability of EBP1 to suppress gene transcription regulated by the cell cycle and prevent cell growth [30, 32]. Furthermore, the expression of EBP1 generates the negative expression of the androgen receptor (AR) and a number of its target genes, thereby inhibiting AR-regulated cell growth [30–33]. RACK1 is a member of the tryptophan-aspartate repeat (WD-repeat) family of proteins. This protein shows significant homology to the β subunit of G-proteins (Gβ). RACK1 facilitates protein binding by adopting a seven-bladed β-propeller structure. Moreover, this protein plays a relevant role in shuttling proteins around the cell, fixing proteins at certain locations and, thus, enhancing stabilization of protein activity. RACK1 cooperates with the ribosomal machinery, with several cell surface receptors and with proteins in the nucleus. As a consequence, RACK1 constitutes a major mediator of various pathways, enhancing numerous phases of cellular function. RACK1 is a scaffolding protein that takes part in the maintenance of intestinal homeostasis protecting the integrity of the epithelial barrier by suppressing the regeneration and proliferation of crypt cells, promotes differentiation and apoptosis and is generated against stress responses [34–36]. Downregulation of both EBP1 and RACK1 may contribute to prevent the hyperplasia in the intestinal tissue that is associated with susceptibility to *E. caproni* infections.

Another striking feature that may be related with alterations in the intestinal epithelium and resistance to infections is the upregulation of annexins 2 and 4 in rIL-25-treated mice exposed to *E. caproni* metacercariae. Annexin is a common name for a family of structurally related proteins that mostly found in eukaryotic organisms both in extra and intracellular environment and bind phospholipids and carbohydrates in the presence of Ca^2+^ [37, 38]. Annexins play a role in the control of cell death and also alters several properties of the membrane such as permeability or anchoring of cytoskeletal elements [39, 40]. These proteins also are related to epithelial cell migration that is a critical event in gastrointestinal mucosal wound healing [41] Furthermore, annexins can act as modulators of inflammation [42]. In the small intestine, the production of annexins appears to be restricted to M cells, playing a role in endocytic transport and membrane scaffolding [43]. Annexins are ligands for phosphatidylserine, which is exposed during cell death. Annexins block posphatidylserine-dependent phagocytosis of dying cells, enhancing its internalization and delivering phosphatidylserine back to the inner leaflet of the cell membrane [44]. Annexins are involved in the repair mechanisms both at tissue and intracellular levels [40]. Upregulation of annexins has been reported in association with resistance to *E. caproni* secondary infections in mice [38]. This was related to the decreased rate of cell death that occurs even though the induction of mitochondrial dysfunction, cellular senescence and elevated oxidative stress [38].

Specifically, annexin 4 appears to play a specific role in membrane repair. Plasma membrane repair mechanisms include internalization *via* endocitosis, or exocytosis as observed from mechanical wounding or exposure to plasma membrane pore-forming agents [45–48]. Therefore, overexpression of annexin 4 due to the exposure to metacercariae of rIL-25-treated mice may contribute to the defense against this parasite infection contributing to the restoration of the intestinal tissue and by its activity as an anti-inflammatory factor. Annexin 2 is a protein that is part of the lipid rafts in the intestinal brush border and is associated with actin filaments mediating in membrane-membrane and membrane-cytoskeletal interactions influencing actin cytoskeletal remodeling through targeting signaling molecules to membrane domains. As a consequence, it plays a crucial role in membrane trafficking and stabilization of membrane-associated protein complexes with the actin cytoskeleton and has been involved in the migration of several types of cells, such as epithelial cells and cell matrix interaction [41, 49–51]. Moreover, annexin 2 induces clustering of specific plasma membrane phospholipids and is involved in lipid domain formation [41]. The lack of annexin 2 would influence RhoA-mediated F-actin reorganization and, consequently, affecting motility of annexin 2 deficient cells [41]. In this sense, our results suggest that the upregulation of both annexins (annexin 2 and 4) could help maintain the epithelial barrier structure during helminth infections.

Quantitatively, the proteins involved in metabolic processes were the most altered in any of the groups studied. A total of 20 of the identified spots (corresponding to 15 different proteins) are metabolic enzymes and most of them were significantly downregulated in mice exposed to metacercariae in presence of rIL-25 with respect to rIL-25-treated mice. Alterations in several proteins implicated in the Krebs cycle (fumarate hydratase and malate dehydrogenase) and in the pentose phosphate pathway (transaldolase and 6-phosphogluconate dehydrogenase). We also observed a reduced expression of glycolytic enzymes including several isoforms of enolase 1B, glyceraldehyde-3-phosphate dehydrogenase and pyruvate kinase PKM, phosphoglycerate kinase 1 and triosephosphate isomerase. This may indicate a mitochondrial dysfunction and a reduction in aerobic metabolism after the exposure to *E. caproni* metacercariae. A similar situation has been described in the ileum of *E. caproni* mice at two weeks post-infection [20]. The decrease of aerobic metabolism was concomitant with a rise in the anaerobic use of glucose, through the overexpression of lactate dehydrogenase. However, Cortés et al. [38] detected a marked downregulation of the production of lactate dehydrogenase in the ileum of resistant secondarily infected mice, suggesting that both aerobic and anaerobic metabolism become impaired as the infection progresses. In contrast, in our study, lactate dehydrogenase was upregulated in the ileum of rIL-25-treated mice exposed to the infection with respect to mice conventionally infected. This might indicate that infection requires an increase in the anaerobic use of glucose to support the high energy demand caused by parasitic infection, both presence/absence of rIL-25 to cover the metabolic demand generated by mitochondrial dysfunction. The consequences of changes in the energy metabolism over the course of the infection is difficult to determine according to our current knowledge. However, it could be of importance to gain a better understanding of the mechanisms activated in the intestine as a consequence of helminth infections.

Several antioxidant and detoxifying enzymes such as peroxiredoxins 1 and 4, glutathione S-transferase and dihydropteridine reductase were also found to be altered. Treatment with rIL-25 induced a marked downregulation of peroxiredoxin 4. This enzyme is a ubiquitously expressed member of the peroxiredoxin family, localized in the endoplasmic reticulum and extracellular space [52]. Peroxiredoxin 4 has a role in the reduction of oxidative stress by diminishing hydrogen peroxide to water in a thiol-dependent catalytic cycle and has also been related to the regulation of nuclear factor kappa B (NF-κB), a key pro-inflammatory transcription factor [53–55]. This supports that the processes related to oxidative stress and cell death are altered in the presence of infection by *E. caproni* independently of the presence and IL-25. IL-25 does not appear to take part in the regulation of the processes related to oxidative stress and apoptosis necessary to maintain intestinal homeostasis. Strikingly, exposure of rIL-25-treated mice to metacercariae caused a downregulation of peroxiredoxin 1 instead of peroxiredoxin 4. Peroxiredoxin 1 plays a key role against reactive oxygen species and antioxidants and in inflammatory responses [56]. The production of this enzyme is upregulated in active ulcerative colitis specimens, and it increases along with the inflammation level in ulcerative colitis regenerative mucosal crypt epithelial cells [57, 58]. Downregulation of peroxiredoxin 1 was observed as a consequence of the cure of an *E. caproni* infection [38]. The reduced production of this enzyme after infection in the presence of rIL-25 may promote crypt-cell proliferation and also induce oxidative stress and ROS-mediated programmed cell death to counteract homeostatic alterations induced by the infection [20, 59, 60].

Infection of rIL-25-treated mice also induced reduction in the production of palmitoyl-protein thiosterase (PPT). Protein thioestherases, or depalmitoylases, participate the depalmitoylation of altered proteins, thus completing a cycle of this reversible post-translational modification [61–63]. Palmitoylation acts as a post-translational “switch” on several proteins providing dynamic control on protein localization or function. Indeed, palmitoylation plays critical roles in protein trafficking and strongly influences the stability of proteins [64–69]. PPT1 is a lysosomal substrate that enters into the lysosome *via* autophagy leading to signaling of several processes related with anabolic and catabolic metabolism in the cell [63, 70]. PTT downregulation is implicated in the disruption of the lysosome-endosomal pathway and in other processes, such as endocytosis, vesicular trafficking, synaptic function, lipid metabolism, neural specification, and axon connectivity. Moreover, it appears to be implicated in susceptibility of cell to apoptotic death, defects in the mitochondrial enzyme activities and adaptive energy metabolism [71]. For this reason, downregulation of PPT after exposure to *E. caproni* metacercariae in the presence of rIL-25 in mice may be related to the role of this protein in processes of regulation of cell death and energy metabolism associated with the maintenance of the intestinal homeostasis. This is supported by the concomitant downregulation of creatine kinase B-type (CKB). This enzyme plays a critical role in energy transduction in tissues with increases in energy demands. The creatine kinase energy system is regulated by hypoxic signaling and can improve creatine metabolism during oxygen deficiency to enhance tissue healing and homeostasis [72]. Impaired Cr/PCr shuttling may contribute to dysregulated mitochondrial energetics and an increased permeability characteristic of inflamed tissue and, consequently, susceptibility to *E. caproni* infection [9, 20, 73].

## Conclusions

Our results indicate that IL-25 and *E. caproni* infection in the presence of rIL-25 induce proteomic changes in the ileum of mice that may contribute to resistance to infection. The main groups of proteins that become altered were those involved in the preservation and healing of the epithelial architecture enhancing the maintenance of the epithelium. Considering our results overall, the maintenance of intestinal homeostasis seems to be essential for resistance to infection. Our study provides new insights into the proteins implicated in the regulation of tissue homeostasis in the presence of rIL-25, a cytokine that is considered as a target factor for the development of resistance to intestinal helminths.

## Supplementary information

**Additional file 1: Figure S1.** Reference image of the 2D-DIGE gel in the experiment comparing infected mice *vs* rIL-25-treated animals, indicating the protein spots identified by mass spectrometry. Green squares indicate downregulated spots and red squares show upregulated spots. Identification details are shown in Table 1.

**Additional file 2: Figure S2.** Reference image of the 2D-DIGE gel in the experiment comparing infected *vs* rIL-25-treated mice exposed to *Echinostoma caproni* metacercariae, indicating the protein spots identified by mass spectrometry. Green squares indicate downregulated spots and red squares show upregulated spots. Identification details are shown in Table 2.

**Additional file 3: Figure S3.** Reference image of the 2D-DIGE gel in the experiment comparing rIL-25-treated exposed to *Echinostoma caproni* metacercariae *vs* rIL-25-treated mice, indicating the protein spots identified by mass spectrometry. Green squares indicate downregulated spots and red squares show upregulated spots. Identification details are shown in Table 3.

**Additional file 4: Table S1.** Details of the computational comparison of protein expression profiles of intestinal epithelial cells isolated from control, control inoculated with rIL-25, infected and infected in presence of rIL-25 mice performed with the EDA module of DeCyder software (GE Healthcare). Manually validated spots displaying significant statistical differences (*P* < 0.01 in one-way ANOVA) are pairwise comparisons of groups. Comparisons are based on the presence or absence of rIL-25, i.e. infected and control against infected in the presence of rIL25, and control inoculated with rIL-25 mice. For each spot in each pair, the fold-change and average normalised volumes (ANV) are shown.

## Data Availability

The mass spectrometry proteomics data have been deposited to the ProteomeXchange Consortium *via* the PRIDE partner repository with the dataset identifier PXD019477.

## Abbreviations

wpi: weeks post-infection
ppi: weeks post-primary infection
pzq: praziquantel
RELM-β: resistin-like molecule beta
IEC: intestinal epithelial cells
HBSS: Hank’s balanced salt solution
FCS: fetal calf serum
RT: room temperature
FDR: false discovery rate
PCA: unsupervised principal components analysis
HCA: hierarchical cluster analysis
GSK3β: glycogen synthase kinase
Wnt: the canonical wingless-related integrator site
SETD7: suppressor of variegation 3-9-Enhancer of zeste-Trithorax domain-containing family of lysine methyltransferases
RACK1: receptor of activated protein C kinase 1
PA2G4: proliferation-associated 2G4
EBP1: proliferation-associated 2G4
Gβ: β subunit of G-proteins
