# Supplementary material for: Ileal proteomic changes associated with IL-25-mediated resistance against intestinal trematode infections
Source: Parasit Vectors. 2020 Jul 2;13:336. doi: 10.1186/s13071-020-04206-y (PMC7331265; doi:10.1186/s13071-020-04206-y)
Supplement: Supplementary file 4 — Additional file 4: Table S1. Details of the computational comparison of protein expression profiles of intestinal epithelial cells isolated from control, control inoculated with rIL-25, infected and infected in presence of rIL-25 mice performed with the EDA module of DeCyder software (GE Healthcare). Manually validated spots displaying significant statistical differences (P < 0.01 in one-way ANOVA) are pairwise comparisons of groups. Comparisons are based on the presence or absence of rIL-25, i.e. infected and control against infected in the presence of rIL25, and control inoculated with rIL-25 mice. For each spot in each pair, the fold-change and average normalised volumes (ANV) are shown. [file 13071_2020_4206_MOESM4_ESM.docx]

**Additional file 4: Table S1.** Details of the computational comparison of protein expression profiles of intestinal epithelial cells isolated from control, control inoculated with rIL-25, infected and infected in presence of rIL-25 mice performed with the EDA module of DeCyder software (GE Helthcare). Manually validated spots displaying significant statistical differences (*p*<0.01 in one-way ANOVA) are pairwise compared between groups. Comparisons are based on the presence or absence of rIL-25, i.e. infected and control against infected in presence of rIL25 and control inoculated with rIL-25 mice. For each spot in each pair, the fold-change and average normalised volumes (ANV) are shown.

| **Spot** | **1-ANOVA *p*** | **CONTROL + rIL-25^a^ vs. CONTROL^b^** | | |
| --- | --- | --- | --- | --- |
|  |  | Fold | *Average Normalised Volumes* | |
|  |  |  | **^INFECTED^** | **^INFECTED + rIL-25^** |
| **533** | 0.003 | **-1.9** | 6.593e+006 | 1.222e+007 |
| **404** | 0.008 | **-1.9** | 1.924e+007 | 3.719e+007 |
| **128** | 0.01 | **-1.2** | 2.318e+006 | 2.873e+006 |
| **527** | 0.012 | **+1.7** | 6.114e+005 | 3.640e+005 |
| **159** | 0.021 | **-1.8** | 5.440e+005 | 9.810e+005 |
| **298** | 0.023 | **+1.3** | 1.224e+007 | 9.537e+006 |
| **478** | 0.027 | **-1.8** | 7.587e+004 | 1.346e+005 |
| **83** | 0.033 | **+1.3** | 1.330e+007 | 1.025e+007 |
| **147** | 0.034 | **-1.5** | 1.597e+006 | 2.424e+006 |
| **311** | 0.035 | **-1.3** | 1.132e+007 | 1.424e+007 |
| **276** | 0.035 | **+1.2** | 8.571e+006 | 7.064e+006 |
| **306** | 0.041 | **-1.8** | 9.212e+005 | 1.668e+006 |
| **88** | 0.042 | **+1.3** | 1.090e+007 | 8.189e+006 |
| **144** | 0.044 | **+1.2** | 1.843e+007 | 1.522e+007 |

| **Spot** | **1-ANOVA *p*** | **CONTROL + rIL-25 vs. INFECTED + rIL-25^c^** | | |
| --- | --- | --- | --- | --- |
|  |  | Fold | *Average Normalised Volumes* | |
|  |  |  | **^CONTROL^** | **^INFECTED + rIL-25^** |
| **184** | 1.23e-004 | **-2.3** | 3.330e+007 | 1.456e+007 |
| **311** | 0.001 | **-1.9** | 1.132e+007 | 6.015e+006 |
| **246** | 0.001 | **-3.1** | 2.679e+006 | 8.543e+005 |
| **156** | 0.001 | **-1.8** | 3.811e+007 | 2.158e+007 |
| **279** | 0.002 | **-2.0** | 2.852e+007 | 1.402e+007 |
| **431** | 0.002 | **-2.0** | 9.898e+006 | 5.033e+006 |
| **144** | 0.002 | **-1.8** | 1.843e+007 | 1.045e+007 |
| **324** | 0.002 | **-2.5** | 4.619e+007 | 1.174e+008 |
| **325** | 0.003 | **+1.6** | 5.771e+006 | 3.503e+006 |
| **177** | 0.005 | **-1.6** | 1.599e+006 | 9.764e+005 |
| **250** | 0.005 | **-1.8** | 1.010e+007 | 5.725e+006 |
| **296** | 0.006 | **-1.8** | 4.568e+007 | 2.514e+007 |
| **243** | 0.006 | **-2.3** | 2.012e+006 | 8.937e+005 |
| **421** | 0.009 | **-1.7** | 1.260e+007 | 7.430e+006 |
| **262** | 0.01 | **+1.8** | 3.102e+006 | 5.524e+006 |
| **271** | 0.01 | **+1.6** | 2.389e+007 | 3.899e+007 |
| **305** | 0.012 | **+1.7** | 8.727e+006 | 1.497e+007 |
| **69** | 0.013 | **-1.7** | 7.426e+006 | 4.276e+006 |
| **166** | 0.013 | **-2.0** | 9.329e+006 | 4.627e+006 |
| **273** | 0.014 | **+2.0** | 9.200e+006 | 1.824e+007 |
| **123** | 0.015 | **-1.7** | 2.575e+006 | 1.475e+006 |
| **91** | 0.016 | **-1.8** | 3.999e+006 | 2.177e+006 |
| **354** | 0.019 | **+2.2** | 9.226e+005 | 1.995e+006 |
| **302** | 0.019 | **+2.2** | 1.354e+006 | 3.041e+006 |
| **226** | 0.019 | **-2.2** | 2.873e+007 | 1.336e+007 |
| **290** | 0.02 | **-2.6** | 1.825e+007 | 7.071e+006 |
| **449** | 0.02 | **-2.1** | 2.798e+006 | 1.355e+006 |
| **214** | 0.021 | **-1.9** | 9.856e+005 | 5.178e+005 |
| **418** | 0.021 | **-1.6** | 4.202e+006 | 2.586e+006 |
| **120** | 0.026 | **-1.4** | 1.221e+006 | 8.893e+005 |
| **133** | 0.026 | **-1.7** | 2.854e+006 | 1.698e+006 |
| **313** | 0.03 | **+1.3** | 1.628e+006 | 2.115e+006 |
| **257** | 0.032 | **-1.8** | 7.507e+006 | 4.278e+006 |
| **154** | 0.036 | **-1.6** | 8.621e+006 | 5.508e+006 |
| **101** | 0.037 | **-1.6** | 3.151e+007 | 1.962e+007 |
| **358** | 0.038 | **-2.5** | 1.394e+006 | 5.580e+005 |
| **63** | 0.041 | **-1.9** | 3.874e+006 | 2.055e+006 |
| **180** | 0.046 | **-1.8** | 1.185e+006 | 6.501e+005 |
| **387** | 0.047 | **-1.8** | 2.973e+006 | 1.687e+006 |
| **260** | 0.049 | **-1.5** | 1.003e+007 | 6.472e+006 |

| **Spot** | **1-ANOVA *p*** | **INFECTED^d^ vs. INFECTED + rIL-25** | | |
| --- | --- | --- | --- | --- |
|  |  | Fold | *Average Normalised Volumes* | |
|  |  |  | **^INFECTED^** | **^INFECTED + rIL-25^** |
| **273** | 0.017 | **+2.4** | 7.494e+006 | 1.82e+007 |
| **291** | 0.021 | **+1.5** | 1.535e+007 | 2.37 e+007 |
| **133** | 0.038 | **-1.4** | 2.394e+006 | 1.69 e+006 |
| **146** | 0.039 | **+1.9** | 1.820e+006 | 3.43 e+006 |
| **148** | 0.041 | **+2.0** | 1.336e+007 | 2.66 e+007 |

^a^ Uninfected control mice inoculated with IL-25 recombinant.

^b^ Uninfected control mice.

^c^ Four weeks post infection with *E. caproni* in presence of IL-25 recombinant.

^d^ Four weeks post infection with *E. caproni*
